# Investigation of a Passive Sensor Array for Diagnosis of Loosening of Endoprosthetic Implants

**DOI:** 10.3390/s130100001

**Published:** 2012-12-20

**Authors:** Cathérine Ruther, Christian Schulze, Andrea Boehme, Hannes Nierath, Hartmut Ewald, Wolfram Mittelmeier, Rainer Bader, Daniel Kluess

**Affiliations:** 1 Department of Orthopaedics, University Medicine Rostock, Doberaner Str. 142, 18057 Rostock, Germany; E-Mails: christian.schulze2@uni-rostock.de (C.S.); andrea.boehme@uni-rostock.de (A.B.); wolfram.mittelmeier@med.uni-rostock.de (W.M.); rainer.bader@med.uni-rostock.de (R.B.); daniel.kluess@med.uni-rostock.de (D.K.); 2 Institute of General Electrical Engineering, University of Rostock, Albert-Einstein-Str. 2, 18055 Rostock, Germany; E-Mails: hannes.nierath@uni-rostock.de (H.N.); hartmut.ewald@uni-rostock.de (H.E.)

**Keywords:** total joint replacement, aseptic loosening, intelligent implant, boundary element method, magnetic induction, loosening sensor

## Abstract

Currently, imaging methods are used to diagnose loosening of endoprosthetic implants, but fail to achieve 100% accuracy. In this study, a passive sensor array which is based on the interaction between magnetic oscillators inside the implant and an excitation coil outside the patient was investigated. The excited oscillators produce sound in the audible range, which varies according to the extent of loosening. By performing several experimental tests, the sensor array was optimized to guarantee reproducible and selective excitation of the sound emission. Variation in the distance between the oscillators demonstrated a definite influence on the quality of the generated sound signal. Furthermore, a numerical design analysis using the boundary element method was generated for consideration of the magnetic field and the selectivity of the oscillators during excitation. The numerical simulation of the coil showed the higher selectivity of a coil with a C-shape compared to a cylindrical coil. Based on these investigations, the passive sensor system reveals the potential for detection of implant loosening. Future aims include the further miniaturization of the oscillators and measurements to determine the sensitivity of the proposed sensor system.

## 1. Introduction

Implantation of a total hip replacement (THR) has become a standard procedure for the treatment of degenerated or fractured hip joints. After resection of the femoral head, a stem is impacted or cemented into the femur. The acetabulum is reamed and a cup is placed into the cavity. The artificial joint is realized by connecting a ball-head to the stem and a liner into the cup. The modular design of modern total hip replacements allows choice of different materials as bearing surfaces in combination with materials of high biocompatibility and enhanced bone ingrowth. Despite more than forty years of clinical experience, complications can still occur which require an exchange (revision) of the THR or some of its components. The main reason for failure is loosening of the stem or the cup [1]. Acute pain requires clarification as to whether the THR is mechanically loose and has to be revised [2]. In orthopedic surgery, each revision of a THR leads to subsequent tissue defects and functional deficits. The sooner the loosening is detected, the better the prognosis for the new implant. However, clinically applied methods of assessing implant fixation with respect to implant loosening are of suboptimal precision, leading to uncertain indication of revision surgery and late recognition of bone defects. In clinical practice, imaging methods, especially radiography, are used to identify the actual condition of fixation, but fail in the aim of achieving 100% accuracy in the diagnosis of slight loosening [3,4]. Thus, vibration techniques were investigated experimentally to assess the structural integrity of endoprosthetic implant systems by evaluating acoustic and structural vibration parameters e.g., damping and resonant frequency [5–12]. Different research studies, including animal experiments, demonstrated a positive correlation between a decrease in the resonant frequency and a decrease of the system stiffness, and therefore an increase in implant loosening [13–16]. The focus of past studies was to obtain vibrational output by exciting the bone-implant-system using an external electrodynamic shaker at the femoral condyles [17–19]. The output signal could be measured with an accelerometer attached to the skin at the prominence of the greater trochanter. Resonant frequencies in the highly sensitive band starting at 2,500 Hz are most sensitive to changes in the biological structure and thus the condition of fixation of the THR [20]. This frequency range is difficult to reach using the shaker, since higher frequencies are reported to provoke pain. Currently, researchers are striving to replace *ex vivo* techniques with novel *in vivo* sensors, such as accelerometers in vibration analysis [21]. For any measurement system that uses electronics integrated in the THR, loss of functionality and degradation during sterilization presents a significant challenge. Besides miniaturized design and biocompatibility, a reliable power supply is an issue in development of *in vivo* sensors. The technology of choice for vibrometry in most studies is wireless powering and data telemetry. Hence, energy can be provided extracorporeally by a primary coil and an *in vivo* secondary coil integrated in the endoprosthetic implant. In order to ensure long-term performance of the *in vivo* electronics, inductivities and capacities have to be chosen carefully. During the design of application specific electric circuits, low operating voltages and extremely low input signal levels appear, resulting in offset voltages and crosstalk from digital to analogue wires [22]. A study with an artificial thigh and a THR equipped with an integrated accelerometer showed damping of frequencies in the highly sensitive band, even though an optimized amplification was applied [23].

**Figure 1.**
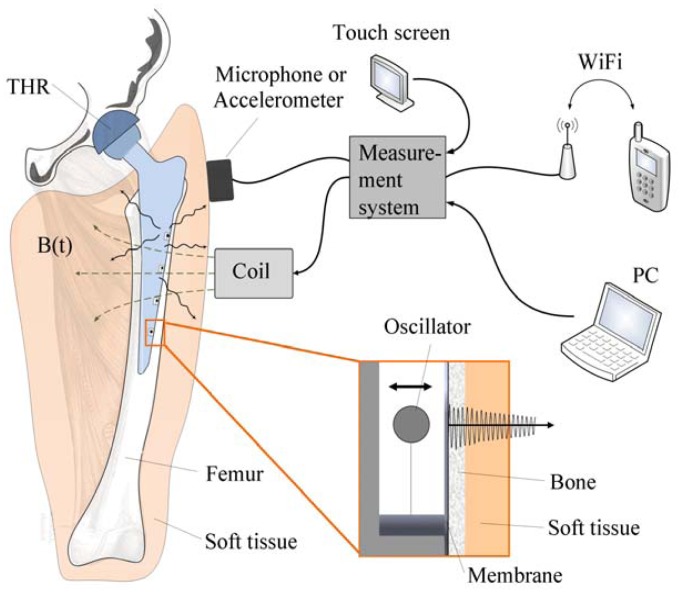
Schematic illustration of the passive sensor array inside a total hip stem.

Due to the aforementioned challenges of implementation of an integrated active system to identify implant loosening, we proposed a passive sensor concept based on magnetic induction [24]. The concept is characterized by an extracorporeal coil, which excites several magnetic oscillators inside the THR to impinge on thin membranes and thus producing a sound and vibration signal (Figure 1). The proposed passive concept consists of several spring-mass-oscillators, which are assembled by a flat spring and a permanent magnetic sphere. This kind of oscillating system can be excited using an excitation coil, due to the polarization of the sphere. The external magnetic field effects the displacement of the oscillator from its neutral position. Hence, the oscillator moves back and impinges on the membrane dependent on its back-spring force. From this central point, sound emission originates due to the excitation of a free oscillation of the membrane-bone system in its bending modes. The resultant structure-borne sound waves propagate as transient elastic sound waves through the adjacent tissue. At the border of the bone-membrane system, changes in the sound characteristics appear dependent on the condition of implant fixation.

The sound emission and vibration of the system can be detected using a structure-borne sound sensor, e.g., an accelerometer or a microphone. To identify loosening at different locations of the hip stem, several integrated oscillators are necessary.

In the present work, the passive sensor array was investigated to guarantee the reproducible and selective excitation of sound emission inside the THR. In the study, one of the most important considerations is how to excite each individual oscillator without influencing oscillators proximal to the excited one, and therefore achieve the highest possible selectivity. In particular, we addressed the question of which parameter adjustments of the extracorporeal coil are most appropriate for the selective excitation of the oscillators. A numerical simulation of two different excitation coil designs, applying the boundary element method, was carried out.

## 2. Methods

### 2.1. Parameter Analysis of the Internal System

The distribution of magnetic field lines of a coil needs to be focussed in order to ensure a selective excitation of the oscillators. Undirected magnetic field lines lead to an activation of several oscillators simultaneously, hence influencing the selectivity of the excitation. The excitation of a single oscillator is of vital importance for avoiding sound interference, especially at one membrane, and hence ensuring highly sensitive measurements. There are different possibilities to achieve a selective excitation of the oscillators in order to measure the sound waves and vibration at each membrane. First, variation of the eigenfrequency of the oscillators ensures differentiation due to providing differently alternating voltages at the excitation coil. The flat spring of the oscillator can be represented as a beam with a constant stiffness and a point mass. By changing the geometry of the flat spring, the bending stiffness as a product of the Young's modulus E and the inertia I of the flat spring can be varied. Thus, the eigenfrequency of every single oscillator can be determined. Since the mass of the oscillator is not varied in the experimental tests, it can be neglected in further considerations. The bending of the beam can be described by the partial differential equation:

(1) $$\ddot{w}+\frac{EI}{\rho A}w=0$$

The eigenfrequency ω_K_ of the free oscillating beam in its different eigenmodes k arises from following equation:

(2) $$\omega_{K}={\left(k^{2}\pi^{2}L\right)}^{2}\sqrt{\frac{EI}{\rho AL^{4}}}$$

**Figure 2.**
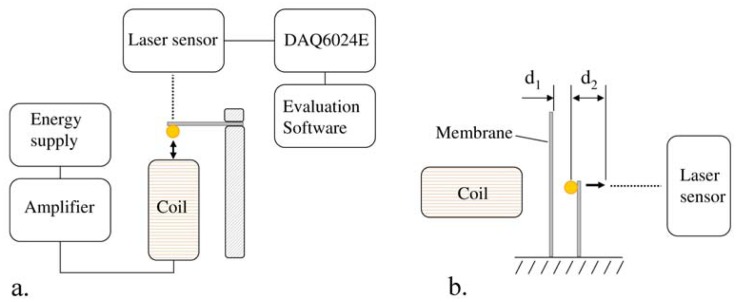
Scheme of the experimental setup. (**a**) Measurement of the eigenfrequency of the oscillator. (**b**) Measurement of the displacement d_2_ of the oscillator after the first impingement, to find the ideal distance d_1_ between the centre of the sphere and the membrane.

Based on this equation, different oscillators were chosen and tested in an experimental setup (Figure 2(a), Table 1). The oscillators were established by fixing a permanent magnetic sphere (Ø3 mm, NdFeB) on a flat steel spring with adhesive. The resonant frequency of each oscillator was measured using an M7L/4 distance laser sensor (MEL Mikroelektronik, Munich, Germany) and a cylindrical excitation coil, where a sweep signal of a function generator between 5 and 500 Hz was induced. The frequency with the highest amplitude equals the oscillators' eigenfrequency and was evaluated. Three oscillators, which had an acceptable difference in the eigenfrequency, were used for further investigations.

A further parameter that can be varied to achieve a selective excitation of the oscillator is the distance d_1_ between the centre of the sphere and the membrane (Figure 2(b)). This is necessary, because an ideally chosen distance d_1_ for the excited oscillator ensures the highest amplitude of membrane oscillations. A further reason is that due to the continuous excitation of the oscillator, the dynamic effects influence the energy of the back spring of the oscillator. Thus, a choice of distance d_1_ that is too small affects the proximate oscillator due to the induced energy of the coil. Hence, the proximate oscillator starts to impinge on the membrane, which should be avoided. Therefore, different distances d_1_ were tested in an experimental setup (2.05, 2.10, 2.15, 2.20 and 2.25 mm).

**Figure 3.**
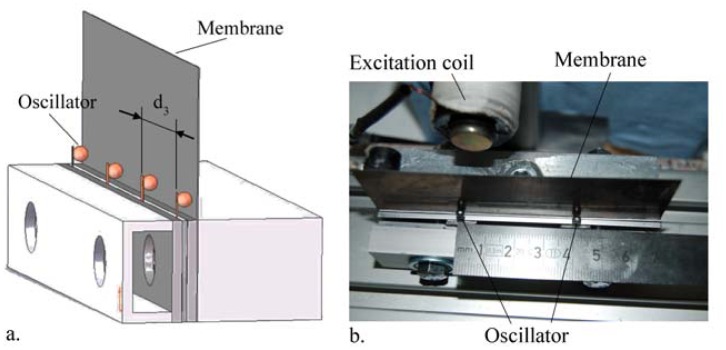
Experimental setup for investigation of the influence on the proximate oscillator during excitation of the desired oscillator by varying the distance d_3_. (**a**) Schematic setup. (**b**) Top view of the experimental setup exemplarily shown for the distance d_3_ = 40 mm, with the excitation of the oscillator on the left hand side.

The last parameter to configure the sensor array in order to achieve a selective excitation is the distance d_3_ between the oscillators (Figure 3). A high number of oscillators inside the total hip stem is desired to ensure the diagnosis over a wide surface. Therefore, the displacement d_2_ of the oscillator proximal to the excited oscillator was tested by fixing the two oscillators in a clamping device with different distances d_3_ (5, 10, 20, 30, 40 and 60 mm).

In order to realize a filigree module consisting of a membrane and oscillator as one component with a high long-term stability, generative procedures are necessary for the cost-effective manufacturing of the oscillators. Therefore, the geometries had to be adapted due to the application of biocompatible Ti6Al4V-alloy. This was done by using the resulting eigenfrequencies of the experiments for the best selectivity and hence varying the geometry of the flat spring until the desired eigenfrequency was achieved. The adaptation of the oscillator is simulated using the modal analysis tool of the Finite-Element-Software SIMULIA ABAQUS CAE Version 6.10 (Dassault Systems, Vélizy-Villacoublay, France). For the simulation, three different oscillator designs were established, based on the results of the experiments. The magnetic sphere was constructed as a point mass in the model. All parts were defined as deformable solids. The base of the membrane-oscillator system was fully encased. After a convergence analysis, 39,700 quadratic tetrahedron elements were used to mesh the parts. In order to calculate the eigenfrequency of the oscillators, the Lanczos solver was used.

### 2.2. Design Analysis of the Extracorporeal System

**Figure 4.**
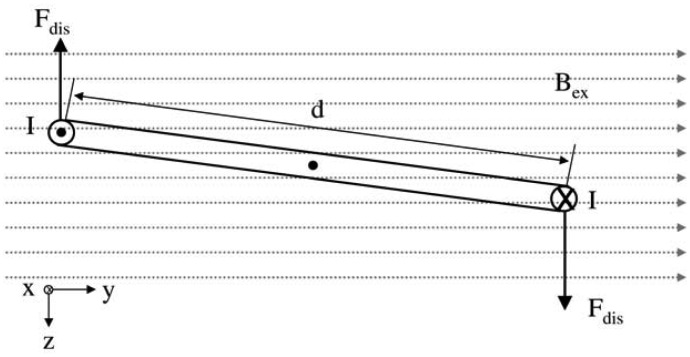
Transfer of the magnetic sphere to a simplified conductor swing model for calculation of the magnetic flux density B_ex_.

During the experiments to test the different oscillator types, we observed that the energy provided by the cylindrical coil over a distance of at least 100 mm would not be sufficient to achieve an excitation of the oscillators e.g., in a human leg. Using a cylindrical coil, the magnetic field lines cannot be concentrated, which is required for the selective displacement of the oscillators. Therefore, an inductive unit with a C-core for application at the patient's leg was designed using numerical simulation based on the boundary element method (FARADAY Version 6.3, Integrated Engineering Software, Winnipeg, MB, Canada). The current I_ex_ and the number of windings N for an optimized excitation C-shape coil could be determined. In order to reduce the complexity of the model, the magnetic sphere can be transferred to the model of a conductor swing, which is positioned in the magnetic field with an imposed magnetic flux density B_ex_ in y-direction (Figure 4). The permanent magnetic sphere provokes a magnetic flux density B_S_ affecting B_ex_. Both magnetic fields have an effect on the displacement force F_dis_ that has to be produced to displace the oscillators. The conductor swing model is used, because the distribution of the magnetic field lines has its highest density in the center of the magnetic sphere. Furthermore, the development of the current I at the surface along the equator perpendicular to the magnetic field lines was assumed.

The magnetization M of the permanent magnetic sphere can be determined using the hysteresis curve. A simplification of the model is possible, if the magnetic field lines of B_S_ are excluded. Without external field lines, the current I can be calculated using M and the diameter d of the magnetic sphere. This was realized by determining the current density J_m_ in a first step:

(3) $$J_{m}=\text{rotM}$$

Following Stokes' thesis, the differential can be converted to an integral equation:

(4) $$\int_{A}J_{m}\text{dA}=\int_{A}\text{rotMdA}=\oint_{A}\text{Mdl}$$

We can assume that the magnetization M is constant over the small integration distance. Thus, M can be placed in front of the integral. As follows, dl can be replaced by the diameter d of the magnetic sphere:

(5) I=Md+external field components

Analogously to the Lorentz force, B_ex_ is calculated. Therefore the force F_dis_ acts on the live conductor, which operates in the external magnetic field. F_dis_ of the oscillator could be calculated using the cross product.

(6) $$d\vec{F}=I(d\vec{l}\times \vec{B})$$

In the next step, it was assumed that the current I, B_ex_ and F_dis_ are linked over the right-hand rule. Current I in x-direction is directed in the conductor swing on the right hand side. B_ex_ affects the y-direction and F_dis_ in z-direction below:

(7) $$\left[\begin{array}{c} {\text{dF}}_{\text{disx}}\\ {\text{dF}}_{\text{disy}}\\ {\text{dF}}_{\text{disz}} \end{array}\right]=I\left(\;\left[\begin{array}{c} {\text{dl}}_{x}\\ {\text{dl}}_{y}\\ {\text{dl}}_{z} \end{array}\right]\;x\left[\begin{array}{c} B_{\text{exx}}\\ B_{\text{exy}}\\ B_{\text{exz}} \end{array}\right]\;\right)$$

After insertion of the vector values, the equation system is reduced to one component of the force-, length- and magnetic field vector:

(8) $$\left[\begin{array}{c} {\text{dF}}_{\text{disx}}\\ {\text{dF}}_{\text{disy}}\\ {\text{dF}}_{\text{disz}} \end{array}\right]=I\left(\begin{array}{c} 0\\ 0\\ l_{x}B_{\text{exy}} \end{array}\right)$$

**Figure 5.**
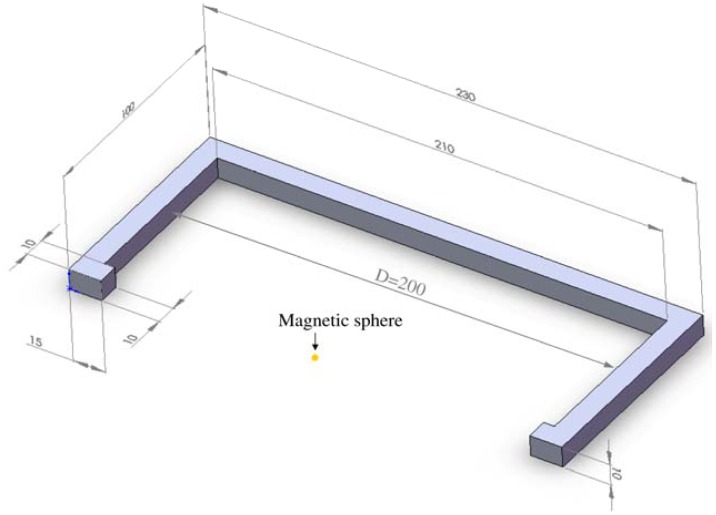
Overview of the dimension of the core for the C-coil.

The design for the coil with a C-shape is aligned with the anatomy of a slim thigh and is characterized by an air gap of 200 mm (Figure 5). The positioning of the coil around the thigh ensures the placement of the magnetic poles of the oscillators in the magnetic field. Hereby, a directed, reinforced and selective displacement of the single oscillator can be realized. The quadratic cross section of both coils of 10 × 10 mm reduces the buckling of the magnetic field lines between both poles.

The simulation model was constructed with blanket planes in a distance of 0.125 mm to the coil core. This distance is equal to the manufacturing tolerance. The basic area of the blanket was assembled from the single areas of the cross sections of the coils multiplied with the number of the coils. In order to produce volume elements with equal volume along the surfaces of the coil core, the area of the equivalent areas was calculated. A volume-current density was assigned to the volume elements. Therefore, this ensured that the current I was consistent over the surface of the coil core. For the modelling of the oscillator, the magnetic sphere was simulated in the centre of the magnetic field. The magnetic sphere was placed at the distance ½ D to both of the poles. For the dimensioning of the inductive unit, the magnitude of the magnetic field at the equator of the magnetic sphere is vitally important. The movement of a magnetic sphere in an alternating magnetic field affords the application of the solver for static frequency independent fields. The coil core is simulated with the properties of iron oxide (Fe_2_O_3_), while for the magnetic sphere the material parameters for NdFeB are assigned (Table 2). For volume elements of the coils and for the surrounding of the system, a relative permeability of μ_r_ = 1 is assumed.

Assuming a consistent distribution at the surface of the coil and A as the area created by the coil cross sections, current I is calculated:

(9) $$I=\frac{\text{JA}}{N}$$

**Figure 6.**
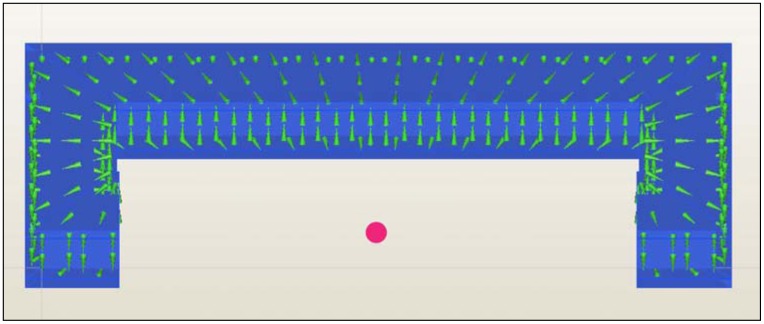
Core of the C-coil with circular direction of the assigned current I_c_.

The entry surface of current I at every blanket face is defined using the right hand rule. The current I automatically flows in every volume element, whereas a consistent distribution of I_c_ along the coil core at all sides of the geometry is ensured (Figure 6). With the same area and an increasing number of windings N, it is assumed that every winding cross section decreases. Accordingly, the current linkage Θ increases, because a higher N per area unit is flowed through by I_c_.

The direction of the magnetization B_S_ of the magnetic sphere is defined in the z-direction. During discretization, the volumes of the model geometry and single surfaces were meshed. For volume elements with assigned I_c_, 3D-brick elements (hexahedral elements) were applied. Furthermore, all surfaces of the coil core and the magnetic sphere were meshed using 2D-triangle elements. The relevant parameters for the dimensioning of the coil are the magnetic flux density B_ex_, especially in the x-direction, the produced displacement force effect F_dis_, the moment which is developed around the y-axis of the magnetic sphere and the displacement of the conductor swing. The y- and z-components of the magnetic field developed a force at the magnetic sphere. This force has no influence because of the directed displacement of the oscillator.

For determination of the optimal range, the coil number of the excitation coil was defined as N = 2,000. Following a convergence study, the components were meshed with approximately 9,000 elements. Validation of the numerical model was carried out by measuring B_ex_ of the existing cylindrical coil using a teslameter (FM210 Teslameter, Projekt Elektronik GmbH, Berlin, Germany). Therefore, the sensor head was positioned in the center in front of the cylindrical coil. Hence, the distance between coil and sensor was varied in steps of 10 mm up to 60 mm. In addition, the effective current through the coil was measured using a multimeter (Agilent Technologies U1242B, Santa Clara, CA, USA).

Different parameters for I and N were simulated to estimate a range in which the excitation of all oscillators could be guaranteed. In order to ensure a selective excitation of the individual oscillators, three different planes were evaluated. The chosen planes are equal to the position of the first three oscillators located distally in the hip stem. Plane 1 (reference plane) is defined with z = 5 mm, plane 2 with z = 19.7 mm and plane 3 with z = 36.5 mm. A reference point in the middle of the air gap is assumed, where the magnetic flux density B_ex_ can be compared. Therefore the quotients of plane 2 to plane 1 and plane 3 to plane 1 were calculated.

## 3. Results and Discussion

### 3.1. Parameter Analysis of the Internal System

**Figure 7.**
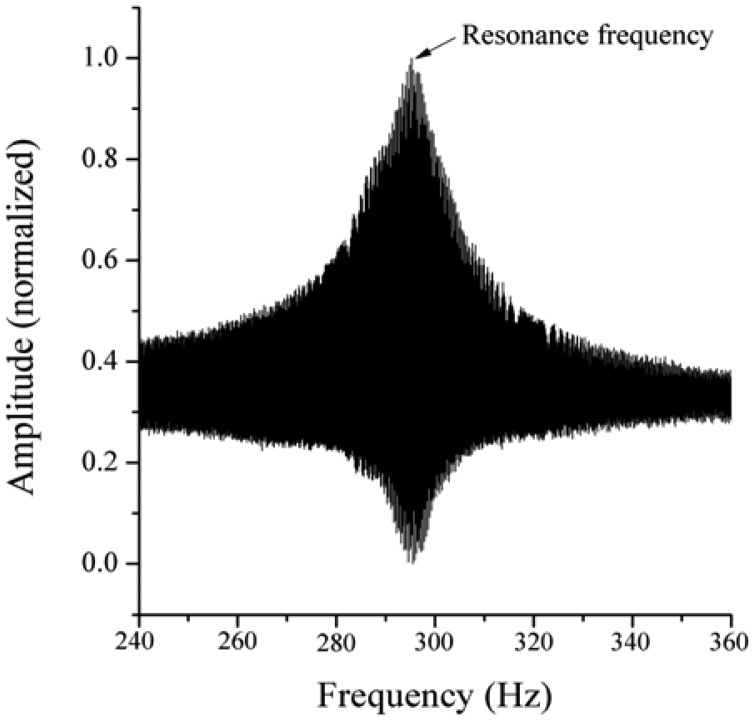
Resonance curve exemplarily for the oscillator type 4 × 2 × 0.05 mm.

Within the eigenfrequency analysis of different oscillators, the highest peak of the resonance curve was determined (Figure 7). The labeled oscillator types in Table 3 were chosen for further measurements and as the basis for the design optimization using the Finite-Element-Method. To guarantee the long term stability, the geometry of the oscillators was chosen to be as small as possible, because additional gaps in the implant cannot be obviated using the proposed concept.

Attaining an excitation of the oscillators with a length of 3 mm was difficult, due to the high energy expenditure, although the distance between coil and oscillator was only 30 mm. Thus, oscillators which exhibit a length of 4 mm were chosen for the passive sensor array in the total hip stem. This was done to keep the gaps in the hip stem as small as possible. According to Equation (1) an increase of the thickness of the flat spring produces an extensive increase in the eigenfrequency of the oscillator. Unwanted oscillation signals of the proximate oscillator are small in the higher frequency range compared to a eigenfrequency below 200 Hz. Therefore, both oscillators above 200 Hz need a smaller difference in the eigenfrequency than the oscillator with a lower eigenfrequency due to the lower energy input required.

Determination of the distance d_1_ between the magnetic sphere and the membrane was a further aim of this study. d_1_ must be ascertained because of the energy influence of the back-spring of the oscillator at the membrane [24]. For realization of the passive sensor array, distance d_1_ of 2.10 mm was chosen, as it supports the highest displacement of oscillator 144 Hz. For oscillator 240 Hz and 286 Hz a distance of 2.05 was chosen (Table 4).

The analysis of the influence of the cylindrical coil on the proximal oscillators showed a high influence on the proximal oscillator for the 144 Hz and the 286 Hz oscillator at distances between 5 and 20 mm (Table 5). This can be explained by the high energy input of the coil on the 286 Hz oscillator due to a forced oscillation in the higher frequency range of the 144 Hz oscillator. In all cases, it could be shown that the displacement of the proximate oscillator is smaller than the distance between sphere and the membrane (2.1 mm), except for oscillator configuration 144 Hz and 286 Hz. For this reason, a distance d_3_ of 20 mm between every magnetic sphere was chosen to reduce the influence of the magnetic field lines as much as possible.

**Figure 8.**
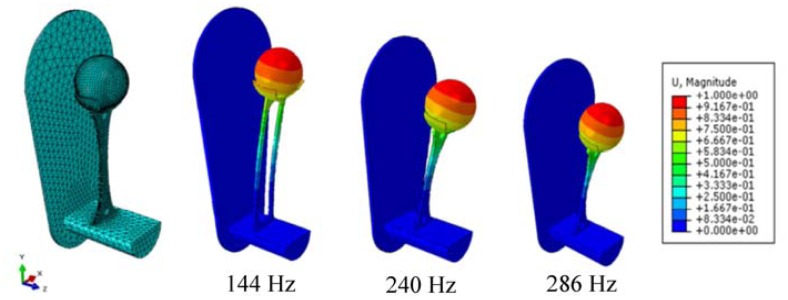
Results of the simulated oscillators creating the geometry variations.

Based on these results, the design optimization of the oscillators for application in a total hip stem was performed using the Finite-element-method (Figure 8). A challenge of the application of oscillators as a passive sensor array is the significant effort required to determine the exact eigenfrequency of every oscillator after the manufacturing process for precise excitation. A further limitation is the substantial effort required to measure the back coupled acoustic signal at every membrane to create a baseline measurement. Further work therefore will include the investigation of the long-term stability of the oscillators.

**Figure 9.**
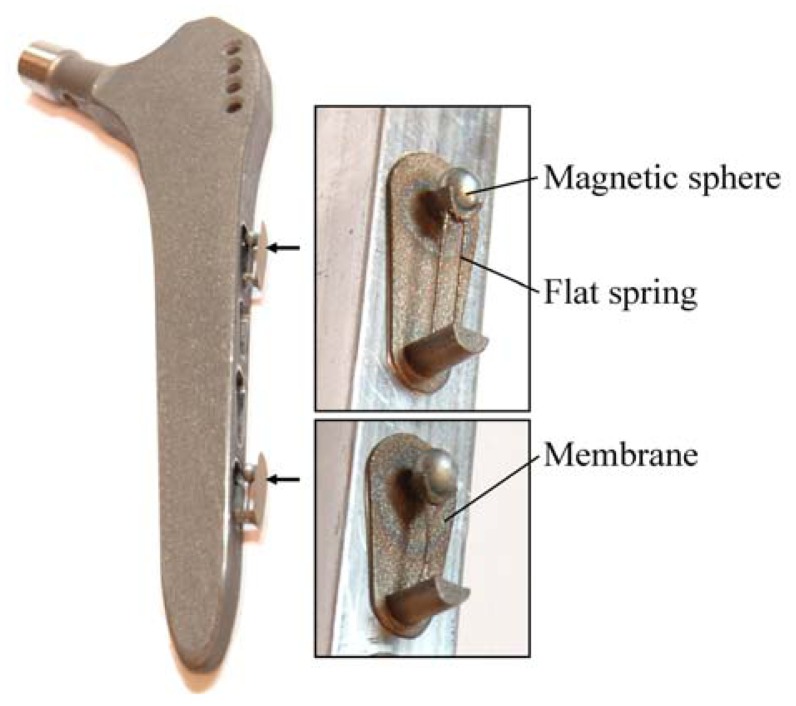
Sensor-equipped demonstrator: Arrangement of the passive sensor array inside a total hip stem.

For the first arrangement of the passive sensor array inside a THR, a custom straight hip stem (Mathys CBH shaft standard, Mathys, Bettlach, Switzerland) was used (Figure 9). Four oscillators were positioned at the lateral face due to an accentuated fixation and bone ingrowths at the distal lateral part of this stem type as a first demonstrator [25]. Hence, distal loosening has a high influence on the stability of the hip stem. The optimized design of the oscillator geometry is manufactured by a generative procedure with rapid prototyping. Here, the oscillators are constructed with membrane as one component (Figure 9).

**Figure 10.**
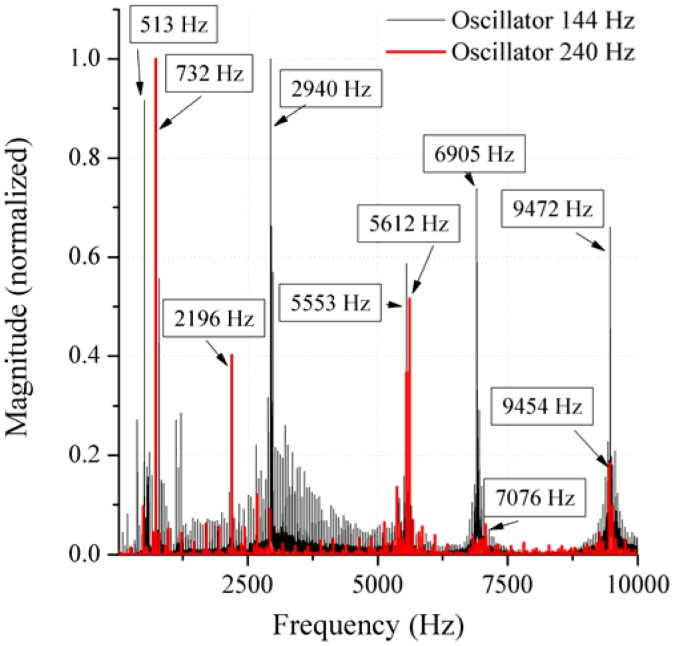
Frequency spectrum of the measurement using the demonstrator and exciting the oscillator type 144 Hz and type 240 Hz.

In order to realize a first test, the demonstrator was proximally fixed in a clamping device and the oscillators were excited with their eigenfrequency and hence recorded by an accelerometer (KS95B, Metra, Radebeul, Germany) positioned at the middle of the total hip stem. The selective excitation of each oscillator could be proven. Furthermore, the frequency spectrum was calculated with a resolution of 0.6 Hz using Fast Fourier transformation (FFT) with a Hanning window of the time signal. The recorded structure borne sound signal consists of many different frequencies, which compose the complete sound spectrum. For this reason, the highest peak of the normalized two-dimensional magnitude-frequency-plot was analyzed. The frequency spectrum shows a difference between the excitation of the different oscillators (Figure 10). For example, in the lower frequency area a difference of 219 Hz could be detected. On the one hand this can be explained by the measurement location, on the other hand, the membrane of the oscillator type 240 Hz is smaller compared to type 144 Hz. Due to the differences in the structure-borne-sound signal, this first measurement applying the new approach has a high potential to detect and locate implant loosening in a total hip stem.

### 3.2. Results of the Design Analysis of the Extracorporeal System

The results of the measured and simulated data of the cylindrical coil revealed an acceptable compliance (Table 6). A resulting maximum deviation of 7.2% can be considered a valid basis for simulation of the C-coil.

**Figure 11.**
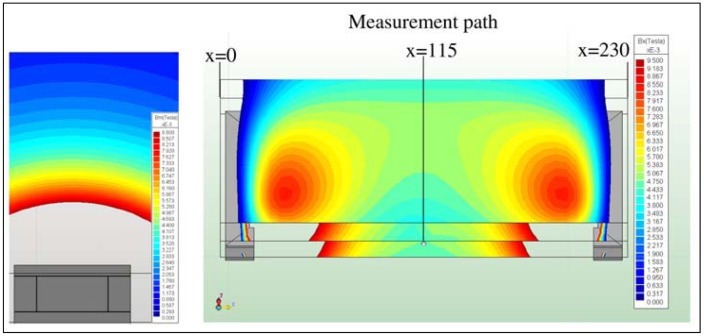
Contour plot of the cylindrical coil (**left**) and the C-coil (**right**) with three evaluated planes. Illustration of the measurement path for evaluation of B_ex_ along the x-axis.

The nominal values of the magnetic flux densities B_ex_ were evaluated. These could be determined by assessing a measurement path along the x-axis (Figure 11). The magnetic field distribution between both poles starts at x = 15 mm and ends at x = 215 mm. The poles are located at x = 0 to x = 15 mm and at x = 215 mm to x = 230 mm. The centre of the magnetic sphere is located at x = 115 mm.

Directly at the poles, a high magnetic flux density of B_ex_ = 240 mT could be identified. A strong decrease of B_ex_ could be visualized at the changeover of the ferrite core to air. For the cylindrical coil, a magnetic flux density of B_exCyl_ = 1.27 mT over an operating distance of 100 mm (consistent to x = 115 mm) was calculated (Figure 11). This corresponds to one half of the magnetic flux density, which is necessary for the displacement of 144 Hz oscillator type. In contrast to the cylindrical coil, the C-coil achieves a magnetic flux density of B_ex_ = 1.79 mT, applying N = 1,000 and I_c_ = 2.24 A (nominal current I_N_ = 3 A). One reason for the lower B_exCyl_ is the absence of the directional characteristics of the cylindrical coil, which is achieved by the C-coil with opposed poles. Excitation of the oscillators using the cylindrical coil can only be realized in the near field at the poles, because the field diverged with increasing distance and lost intensity. It should be mentioned that the external field components are not considered during the transfer of the magnetic sphere into the conductor swing. These components have an influence on the virtual current I which is developed at the surface of the magnetic sphere, producing an increase. Hence, the magnetic flux density that is used for the displacement of the oscillator decreases. In this case, a conservative consideration of the magnetic field is given. Furthermore, the hexahedral profile of the C-coil compared to the cylindrical coil is apparent, which can be explained by the directional characteristics and the more homogeneous distribution of the magnetic field lines between both poles.

**Figure 12.**
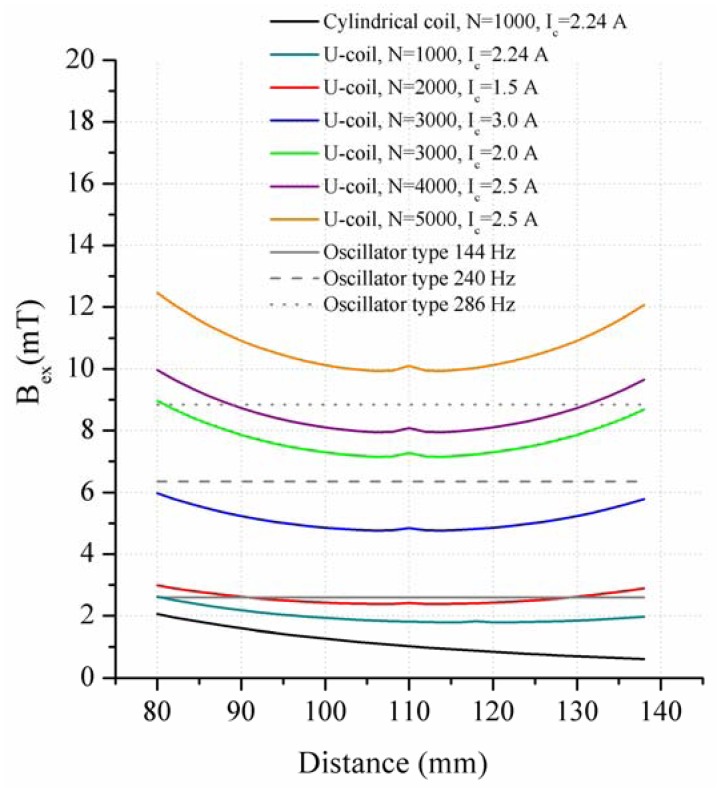
Comparison of the generated magnetic flux densities of the C-coil using different windings N and currents I_c_ in comparison with the calculated values of the different oscillator types.

Based on these results, different parameters of N and I_c_ were simulated in order to get the range over which all oscillator types can be excited (Figure 12). Due to better directional characteristics of the C-coil, it is possible to produce an adequate magnetic flux density with relatively small currents of I_c_ = 2.5 A in the centre of the air gap. The limitation of I_c_ to 3.0 A referred to the application in an electric circuit, in which the excitation coil has to be integrated. Important values of B_ex_ between x = 80 mm and x = 150 mm are relevant for the desired displacement force of the oscillators. Within this range B_ex_ lies between 2.42 mT and 10.1 mT. The numerical values of B_ex_ ensure a displacement of the 240 Hz and 144 Hz oscillator types inside the air gap simulating N = 3,000 and a current of I_c_ = 2.0 A. Figure 12 shows that the 286 Hz oscillator type can only be displaced by a C-coil configured with N = 5,000 and I_c_ = 2.5 A. The numerical values of B_ex_ are determined for the equator of the magnetic sphere. In case of a higher permeability, the relations do not change significantly.

**Figure 13.**
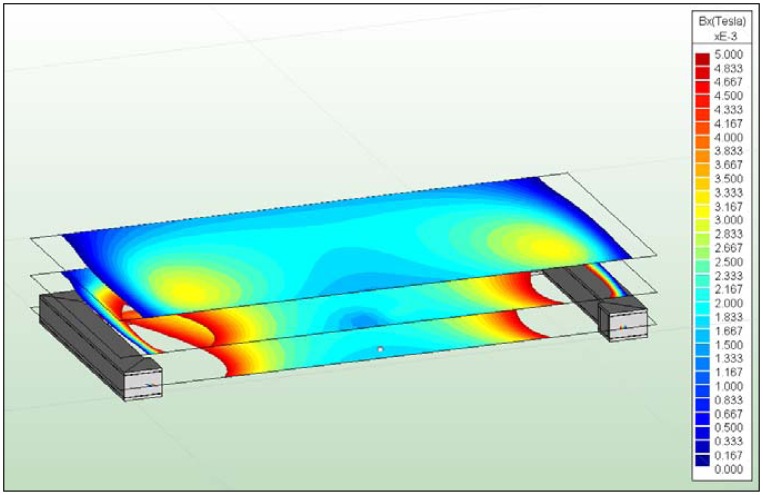
Contour plot of the distribution of B_ex_ in three different planes of the magnetic spheres using a winding number of N = 1,000 and a current I_c_ = 2.24 A.

For the estimation of the selectivity, plane 2 and plane 3 are compared to plane 1 (Figure 13, Table 7). It could be shown that B_ex_ of plane 1 to plane 3 is attenuated by 13%. Upon comparison of plane 1 and plane 2, an attenuation of the intensity of 5% was carried out. For the realization of an optimized excitation coil, the magnetic field was charged with a security factor of 0.06, without losing the advantage of a selective excitation.

Despite an increase of 6% in plane 1, B_ex_ in plane 3 is below the displacement of the oscillator of the desired magnetic field. The selectivity can be reached by a variation of the excitation frequency, and is ensured for the excitation in plane 1, 2 and 3 (Figure 13). In contrast to the cylindrical coil, the C-coil allows the displacement of the oscillators over a distance of 100 mm. Using the cylindrical coil over this distance, an excitation is not possible. This means that with the C-coil a magnetic flux density of B_ex_ can be generated, which is 719% of B_ex_ of the cylindrical coil. The influence of the air gap on B_ex_ is obvious, and the magnetic field lines describe a curve between both poles with decreasing gap size. This is a disadvantage for the positioning of the coil at the patient's leg and hence for the selectivity of the oscillators. A further aspect under consideration is the cross section of the simulated coil. A shape with circular cross section is always the best choice regarding a loss of magnetization. Due to the manufacturing process using a ferrite core, the coil could only be realized with a hexahedral cross section.

### 3.3. Benefits

Over time, several approaches for diagnosing loosening to total hip replacements have been investigated. It was shown that mechanical oscillations of the implant-bone-system could be excited with an electrodynamic shaker [12,17–19]. A challenge which cannot be neglected is the influence of the shaker on the patient's knee, due to the manifestation of pain and a defined contact between shaker and skin. Application of the proposed passive sensor array will not be painful, because of the low oscillator mass and small excitation frequencies. Nevertheless, electronic devices using telemetry and accelerometers that were inserted in the total hip stem in former studies did not withstand the standard sterilization process [21,23]. The simple and robust passive sensor array has a high resistance to sterilization and impaction during implantation. Due to the integration of several oscillators along the total hip stem, the presented approach is more sensitive to implant loosening, because locations of implant loosening can be diagnosed from the read-out of different sounds produced by the impingement of the oscillators at the membranes. This was not possible using former approaches.

Hao *et al.* presented a further concept that included a measurement system for investigation of implant migration and micro movements [26]. This is realized with two coils that are coupled over a core. During the implantation; the core is in contact with the total hip stem in order to detect a measurable voltage. For this concept; an active electronic device is inserted in the femur. The way that the system is implanted is significant; since imprecise measurements during positioning cannot be compensated for by the calibration process [26]. In comparison; the proposed passive sensor array is independent from the implantation process of the total hip stem. Furthermore; the sensor array only needs one postoperative baseline measurement.

### 3.4. Limitations

Due to the integration of the passive sensor array at the lateral side of the hip stem, the long-term stability of the total hip stem was considered in Finite-Element-Analyses. Unpublished data about maximum stresses and static strength show promising results. A static analysis showed that stresses were in an acceptable range if the cavities for the oscillator-membrane-component are small enough. The feasibility has to be proved by a long-term fatigue strength test. Thus, the oscillators may need to be further miniaturized, taking into consideration the production of a reasonable acoustic signal, which should be strong enough to be detected by a microphone or at the skin by an accelerometer. Further miniaturization must be considered, although the manufacturing process is limited due to the thickness of the membrane and the spring. Thus, the eigenfrequency of the oscillator increases and hence skin effects in the coil can appear. This effect results in higher currents, which cannot be produced to displace the oscillators. Another point for consideration is the measurement system, which has to be adjusted to the resonant eigenfrequencies of the oscillators. The functionality of acoustic sound and structural vibration detection was demonstrated on overdimensioned models [24] and in *in vitro* studies using porcine forelegs [27,28]. Further work requires the determination of the sensitivity of the passive sensor array to implant loosening.

Furthermore, an additional stand for the coil to achieve a precise excitation should be considered. This could be achieved in further steps by varying the magnetic field strength of the magnetic spheres in order to determine the location of each individual oscillator by applying sensors which are capable of measuring the magnetization at every location.

## 4. Conclusions

In the present study, a novel passive sensor array was investigated for implementation in endoprosthetic implants in order to identify implant loosening. The main focus of the work was to achieve a reproducible and selective excitation of sound waves inside the implant. A basic sensor array was constructed and tested for integration in the total hip stem. Furthermore, eigenfrequencies of the oscillators were determined to identify three oscillator types with a high difference in order to ensure the excitation selectivity. Optimization of the inductive extracorporeal unit of the measurement system using finite-boundary simulation for a cylindrical and C-shaped coil revealed the highest selectivity by exciting the oscillator using a C-shape. The presented simulations showed how the coils behave during application and how the magnetic field characteristics are developed. With the comparison in the different planes and the resulting attenuation of the intensity of the magnetic field, an appraisal of the selective excitation of the different oscillator types is possible. The results show that due to the variation of the oscillator geometry, the oscillators can be excited selectively. The parameters for the C-coil have to be chosen very precisely.

Based on these investigations, the passive sensor system reveals the potential for detection of implant loosening. The next steps include the further miniaturization of the oscillators and tests of functionality of the demonstrator. Furthermore measurements to determine the sensitivity of the proposed sensor system will be realized including *in vivo* studies and in clinical setting.

## Figures and Tables

**Table 1.** Geometries of the tested oscillators.

**Length (mm)**	**Width (mm)**	**Thickness (mm)**

3.00	2.00 and 1.00 each length	0.05
4.00		
5.00		
6.00		
7.00		

7.00	2.00	0.10

**Table 2.** Overview of the material properties used for the simulation of the excitation coils.

**Material**	**Relative Permeability μ_r_**	**Magnetic Remanence B_r_ (T)**	**Coercive Field Strength H_C_ (A/m)**	**Conductivity ς (S/m)**
NdFeB	-	1.3	923,098.670	833,333.330
Fe_2_O_3_	650	0.15	35	>1 × 10^−5^

**Table 3.** Results of the eigenfrequency measurement as mean value (f) and associated standard deviation (sd) of different oscillators (bolded values are the parameters chosen for further investigations).

**Geometry (mm)**	**f (Hz)**	**sd (Hz)**

3 × 2 × 0.05	367.00	3.65
**4 × 2 × 0.05**	**285.62**	**0.88**
5 × 2 × 0.05	219.94	0.38
**6 × 2 × 0.05**	**143.60**	**1.06**
7 × 2 × 0.05	124.16	0.20
3 × 1 × 0.05	361.93	3.00
**4 × 1 × 0.05**	**240.14**	**0.43**
5 × 1 × 0.05	122.57	1.19
6 × 1 × 0.05	114.54	0.28
7 × 1 × 0.05	71.68	0.33
7 × 2 × 0.10	229.25	0.56

**Table 4.** Results of the measurement of the displacement d_2_ as the highest amplitude to configure the optimal distance d_1_ between the centre of the magnetic sphere and the membrane (bolded values are the distances chosen for further investigations).

**d_1_ (mm)**	**Oscillator 144 Hz**		**Oscillator 240 Hz**		**Oscillator 286 Hz**	

	**d_2_ (mm)**	**sd (mm)**	**d_2_ (mm)**	**sd (mm)**	**d_2_ (mm)**	**sd (mm)**

2.05	0.341	0.003	**0.291**	**0.003**	**0.224**	**0.003**
2.10	**0.721**	**0.005**	0.271	0.003	0.215	0.003
2.15	0.623	0.003	0.265	0.002	0.213	0.005
2.20	0.568	0.001	0.231	0.003	0.161	0.003
2.25	0.324	0.003	0.211	0.003	0.119	0.003

**Table 5.** Results of the measurement of the displacement d_2_ of the influenced oscillator proximal to the excited oscillator at different distances d_3_.

**d_3_ (mm)**	**144 Hz–240 Hz**		**144 Hz–286 Hz**		**240 Hz–286 Hz**	

	**d_2_ (mm)**	**sd (mm)**	**d_2_ (mm)**	**sd (mm)**	**d_2_ (mm)**	**sd (mm)**

5	0.196	0.003	1.05	0.005	0.146	0.001
10	0.123	0.002	0.865	0.001	0.132	0.002
20	0.072	0.003	0.243	0.003	0.113	0.001
30	0.057	0.003	0.031	0.001	0.094	0.002
40	0.032	0.001	0.015	0.002	0.075	0.002
60	0.012	0.001	0.002	0.001	0.054	0.001

**Table 6.** Results of the validation of the numerical model of the cylindrical coil using an effective current of 2.24 A.

	**B_ex_ (mT)**					

**Distance to coil**	**10 mm**	**20 mm**	**30 mm**	**40 mm**	**50 mm**	**60 mm**

**Experiment**	60.1	30.2	19.1	12.8	8.8	6.3
**Simulation**	55.8	30.3	19.5	12.4	8.5	6.5

**Table 7.** Results of the magnetic flux density in the center of the air gap using different parameters.

**N**	**I_c_(A)**	**B_ex_ Plane 1 (mT)**	**B_ex_ Plane 2 (mT)**	**B_ex_ Plane 3 (mT)**

2,000	1.5	2.42	2.31	2.12
3,000	2.0	4.85	4.62	4.23
	3.0	7.27	6.94	6.35
4,000	2.5	8.08	7.71	7.05
5,000	2.5	10.10	9.63	8.82
